# Death and Happiness: Exploring the Temporalities of the Meditated Death and Everyday Life in Tibetan Buddhist Practice of *Tukdam*

**DOI:** 10.1007/s11013-025-09914-7

**Published:** 2025-05-21

**Authors:** Tenzin Namdul

**Affiliations:** Earl E. Bakken Center for Spirituality & Healing, University of Minnesota, 420 Delaware St SE, MMC 505, Minneapolis, MN 55455 USA

**Keywords:** Death, *Tukdam*, Tibetan Buddhism, Tibetan medicine, Meditation, India

## Abstract

Although *tukdam*—a meditative state entered through various practices resting in extremely subtle consciousness while dying—is seen to only be achieved by adept practitioners, the philosophy and psychology that underpin *tukdam* inform Tibetan communities beyond just accomplished adepts and frame the very way death and dying is conceived. Based on an 18-month ethnographic study, this article explores the significance of death as a Tibetan Buddhist cultural-reference that offers a moral heuristic ground for adaptive methods in transforming orientations to self and others and in cultivating compassion and resilience. Tibetan Buddhist practitioners emphasize a strong correlation between a true understanding of self and sustained happiness. This article thus asks a dual conceptual question: (1) Why do Tibetans believe that meditating on death is the key to experiences of well-being in their day-to-day life? (2) What is the relation between the temporalities of the meditated death and that of the day-to-day life? Furthermore, the article proposes that Tibetan Buddhist practices that culminate in *tukdam* symbolize the way death and dying is assumed to be approached more broadly beyond advanced practitioners, and thereby, provides a cultural model for an “ideal” death that guides approaches to dying for oneself and others.

## Introduction

It has been almost two decades, and I still remember it vividly. It was a hot and humid evening on September 24, 2008, in Mundgod Tibetan Settlement, where two of the largest Tibetan Buddhist monastic universities—Drepung and Gaden—are located in South India. My colleague and I nervously unpacked all the scientific equipment—electroencephalograph (EEG), electrocardiogram (EKG), thermal camera, among others—and fiddled with the myriad of electric cords. We were at Drepung Loseling Monastery to implement a scientific study on a senior monk who had been clinically dead for more than a week, yet his body had remained intact because he was in the state of meditation called *tukdam* (Tib. *thugs dam*). In other words, the senior monk, Lobsang Nyima Rinpoche,^1^ who was the hundredth Gaden *Tripa*,^2^ was engaged in a contemplative technique whereby he maintained complete control over the physiological decay of his body via regulation of his subtlest consciousness (*shin tu phra ba'i shes pa*). We were there to scientifically investigate what was happening during this process by employing these neurophysiological measures (see Lott et al., 2021; Tidwell et al., 2024, for the initial null finding results and delayed decomposition changes among deceased practitioners from the broader study).

Tanya Zivkovic (2010), in her ethnographic account^3^ of a Tibetan Buddhist lama’s biography, describes his *tukdam* as a way of displaying control over death and “both an indication and outcome of an elevated spiritual status” (175). Zivkovic explains *tukdam* as a state of meditation where accomplished practitioners “impede physical flaccidity ordinarily preceding rigor mortis, retain a meditative state, suspend the processes of decomposition, maintain warmth in the body, produce a pleasant scent, transmogrify bones into images of Buddhist deities and manifest rainbows” (176; also see Lott et al., 2021; Tidwell, 2024, for different characteristic signs of *tukdam*).

Lobsang Rinpoche’s body had no signs of decomposition, putrefaction, or discoloration, and was still warm. Besides being anxious about properly setting up the equipment since it was our first study participant, I was a little overwhelmed by everything I was witnessing—an advanced practitioner who was still in full meditation posture whose complexion and positioning looked every bit alive amidst a group of rather excited monks completely engrossed in their roles. Yet he had been clinically dead for over a week. I could not help myself thinking this is very different from deaths I had observed in the past and wondered: Why would a Buddhist practitioner decide to practice meditation at the specific time of “dying”? How is it different from entering meditation while alive? What is the purpose of death in relation to entering *tukdam*? And importantly, what effect does it have on the way such practitioners and other members of society choose to die, and care for the dying?

The article thus explores the cultural significance of death for Tibetan Buddhists and its role as a moral heuristic ground to transform orientations toward self and others. Tibetan Buddhist practitioners use death to contemplate two fundamental Buddhist concepts: impermanence (*mi rtag pa*) and the interdependent nature (*rten ’brel*) of phenomena. While contemplating impermanence provides an alternative perspective of viewing death as an integral part of life, contemplating interdependence helps one deconstruct the conceptual-experiential importance habitually placed on and distortedly reifying our sense of self. This technique, according to Tibetan monastics I interviewed, generates what they call “heart power” (*snying stobs*), also described as “mental strength,” offering a spiritual opportunity at the time of dying and a buffer against fears of death. Such acquired psychological attributes aid to strengthen one’s resilience and generate compassion, in addition to Buddhist ontological knowledge of death and dying (Coleman, 2025).

I realized that, given the inextricable links among Tibetan Buddhism, Tibetan medical practice, and Tibetans’ way of life in general, *tukdam* holds an important place in any investigation of death in a Tibetan cultural context. The philosophy and psychology that underpin *tukdam* inform the core components of cultural models that deal with death and care of dying people among Tibetan populations. *Tukdam*, therefore, not only animates Buddhist philosophical concepts, but also inspires and motivates cultural practices—including medical practice, focused on leading a meaningful life and providing ethical care for a dying person. I propose that the Tibetan Buddhist state of *tukdam* symbolizes the way death and dying is assumed to be approached, and thereby, encourages facilitating a culturally appropriate or an ideal death for oneself and others in the Tibetan cultural sphere. With this background, I intend to use *tukdam* as a framework that provides a fundamental structure to Tibetan cultural notions and practices of death and dying.

Interweaving my initial encounter with* tukdam* in 2008 and 18 months of ethnographic study in 2016 and 2017 among Tibetan refugees in South India in which I observed several additional cases of *tukdam*, I explore how a particular cultural significance ascribed to death motivates a unique epistemology of death. I outline the basic framework for this epistemology in this article. I show how such an understanding of death propels an in-depth self-investigation of death and the process of dying that implicates transformations in body, mind, and consciousness.

## First Encounter with *Tukdam*

Two days before we were sitting by the side of Lobsang Rinpoche in the state of* tukdam*, I was preparing for a health outreach trip to Kathmandu, Nepal. It was a bright fall morning after the last stretch of Dharamsala’s notorious monsoon when I got a phone call from the director of Men-Tsee-Khang, the Tibetan Medical and Astro-science Institute headquartered in northern India, relaying information from His Holiness the Dalai Lama’s office. The director told me I need to urgently go to Drepung Monastic University in South India to attend a senior monk who had died a week prior and was still residing in the state of *tukdam*. Drepung Monastery, one of the three largest Tibetan Buddhist academic monasteries reestablished in India, is in Mundgod, 1,450 miles south of Dharamsala, in Karnataka State.

A year earlier, our research team had been trained in data collection and the handling of relevant equipment, such as EEG for recording electrical activity of the brain, EKG to read the electrical activity of the heart, and thermography to record body temperature, for the study. However, we had not had an opportunity to assess an actual subject until this moment. We were excited to finally have our first study participant. I had sparse knowledge about *tukdam,* and the practices engaged to enter it at that time, and I was a little skeptical as well. However, I was curious to learn more about *tukdam*. After canceling my trip to Kathmandu and frantically working with a local travel agency to book a flight that morning, I, along with a Tibetan colleague trained in Western medicine, flew to Mundgod. We landed at the small airport in Hubli and saw an elderly Tibetan man waving at us by the exit gate. Without saying anything, he smiled, grabbed our oversized baggage trolley, and said, “Everyone is waiting for you two.”

Drepung Monastery is an hour and a half drive from Hubli airport. We could hear prayers and see monks sitting in a group inside the compound as we drove toward the gate of a small two-story house where Lobsang Rinpoche’s meditative decedent body resided. In the house, another group of monks were reciting prayers, while others were busy cleaning butter lamps and preparing food in a make-shift kitchen for the monks reciting prayers. We were greeted enthusiastically by Choesang and Loden, the two main attendants of Lobsang Rinpoche.

Given that it was our first study participant, and that he had been declared clinically dead at the hospital, I was not sure how the upcoming hours and days of our time there would unfold. The attendants told us it had been nine days since their teacher entered *tukdam*. They quickly ushered me and my colleague into a small room on the second floor of the house. An older monk was already in the room, who Loden introduced to us as Jampa Rinpoche. Jampa Rinpoche, probably in his late sixties, had a relaxed, calm demeanor as he welcomed us and instantly made us feel comfortable. Jampa Rinpoche was well respected at the monastery and especially revered for his vast experience in supervising those in the state of *tukdam* meditation. He had been observing Lobsang Rinpoche since the *tukdam* state commenced.

The small room was sparsely furnished with just a bed on which Lobsang Rinpoche was lying by the window overlooking the backyard. There was an altar with the Dalai Lama’s portrait and a couple of blockprint Buddhist texts wrapped neatly in saffron cloth. Lobsang Rinpoche’s body was covered with a richly yellow-colored cloth as well. It was hot inside the room even though a ceiling fan was on. While I surveyed the room to find an appropriate spot to set up our equipment, I looked at Lobsang Rinpoche’s face and was riveted by what I saw: he looked like he was just sleeping. As we uncovered his body, I observed that there were no signs of decomposition nor any foul odor despite him having been declared dead more than a week prior.

As we were getting ready Choesang whispered to us that it would be more appropriate if we could set up our investigative devices, including the electrodes that would be plastered to his head and chest and a thermal camera that detects core body temperature, in the evening after the group prayer session because some of the senior monks who had been Lobsang Rinpoche’s students were feeling uncomfortable. We readily agreed with his suggestion as it is usually culturally almost impossible to get access to a monastic in such a meditative condition. If it was not for the Dalai Lama’s keen interest in scientifically studying *tukdam* in order to make the occurrence of and implications for the meditative state known to a wider population, it would have been extremely difficult to convince monks to let us study someone engaged in *tukdam* practice.

## Extraordinary Experiences

For the next fourteen days, my colleague and I had the privilege of being around Lobsang Rinpoche’s body as we collected data—hooking him up to electrodes for EEG and EKG and setting up a thermal camera to measure the degree (or absence) of infrared radiation emitted by the body every evening and removing the devices early in the morning. During this whole process of observing and collecting data, I witnessed a few things which I would have never believed prior to the experience. When I surveyed the room while initially setting up our machines, I noticed a small jar half-filled with water and a twig with a few leaves placed in it on a small stool next to the altar.

The plant looked like a small twig plucked from one of the mango trees which were abundant across the backyard of the house. I saw that one of the monks would get a fresh clipping every morning or every other day. Then one morning a monk who came to change the clipping sat there rather amused and kept on looking closely at the plant. I noticed him but did not ask anything until the monk looked at me and my colleague and told us there was no way the twig could be growing in the jar. He pointed at leaves growing out of the twig and excitedly blurted, “Look at these leaves!” as if there were all precious gems hanging from the twig. From that morning, the small twig in the jar kept developing new growth and the monk responsible for changing the plant would come, look at the twig, and leave it as it was. The attendants and other monks related the unusual growth of the plant to the power of Lobsang Rinpoche’s meditation and could not stop talking about the marvel of the meditative power.

A couple of days later, my colleague and I were embarking on our daily routine of heading over to Lobsang Rinpoche’s residence from our guest house to detach the research devices. It was around 6:30 am and some of the younger monks were already in the kitchen preparing breakfast. As I opened the door to Rinpoche’s room, I smelled a fragrance which I had not detected prior. I knew it was not only me since my colleague and I looked at each other validating one another’s experience without saying anything. It smelled like an amalgam of flowers, and the fragrance became more and more pronounced with each passing day. We would smell the fragrance every morning when we entered the room to remove all the devices; and the scent would gradually fade away through the course of a day. I initially thought one of the attendants might have sprayed room freshener, but this was not the case. It was quite clear that the fragrance was emanating from Lobsang Rinpoche’s body. Jampa Rinpoche later told us that such a fragrance is called *tsültrim kyi dri* (*tshul khrims kyi dri*), meaning a “fragrance of pure morality or ethical discipline.”

Manifestation of such a fragrance during *tukdam*, I was told, is associated with a practitioner’s dedication toward one’s commitment to tantric practice as well as commitment to one’s students. However, it is not customary that such a sign would be present for every practitioner in *tukdam*. I also realized during my later fieldwork that even though some practitioners who enter the state of *tukdam* do not produce a fragrant odor, they do not produce a foul odor either.

**Fig. 1:**
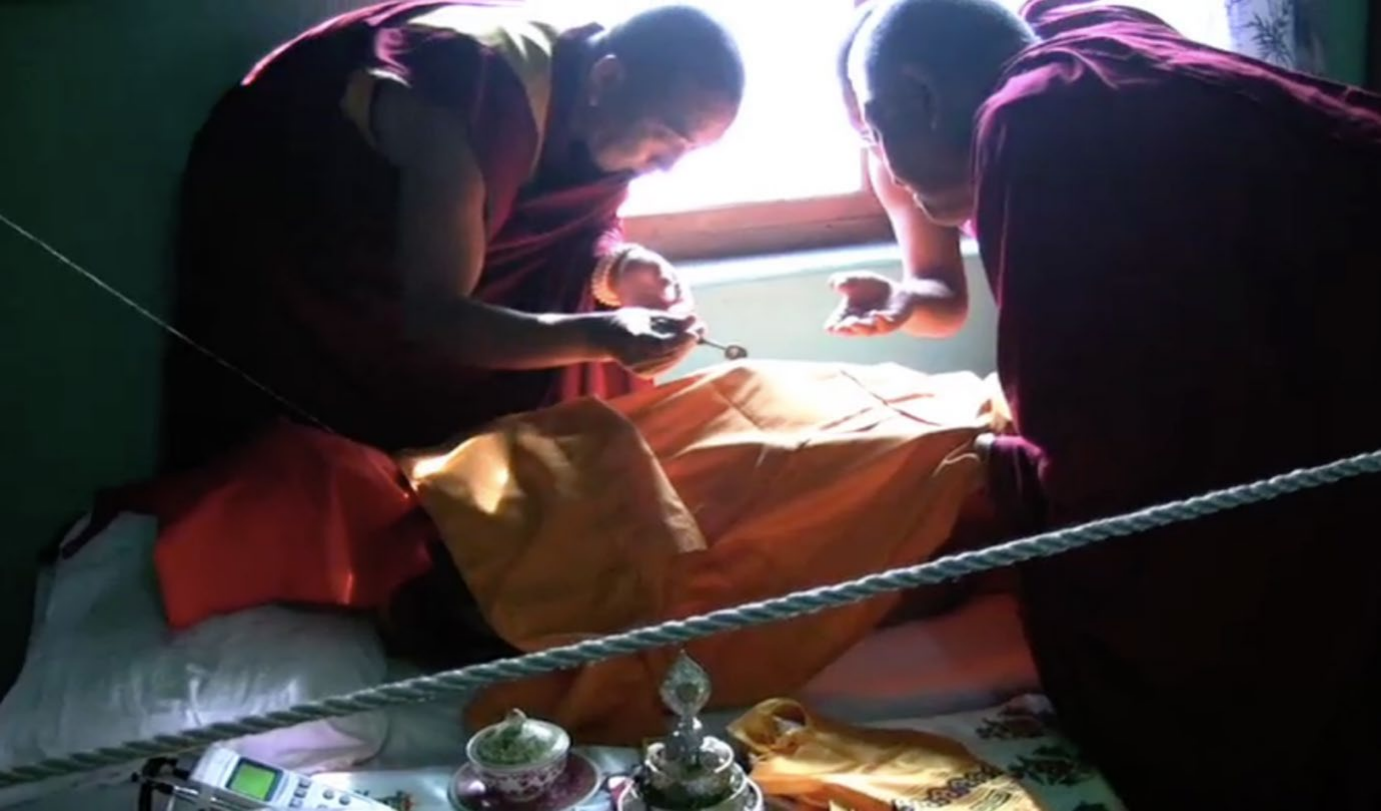
Jampa Rinpoche and Choesang collecting *ringsel* from Lobsang Rinpoche’s body

Another incident that astonished me was seeing pearl-like beads start to appear on Lobsang Rinpoche’s body. One morning as we were getting ready to unhook all the devices, Jampa Rinpoche said, “We need to be careful today as it looks like we have *ringsel* on Lobsang Rinpoche’s body.” Jampa Rinpoche and Choesang carefully scanned the cloth placed on Rinpoche’s body, using a particular spoon made of silver to collect the pearl-like beads and put them in a container (see Fig. 1). *Ringsel* (*ring srel*) are also known as sacred relics and are seen to reaffirm a practitioner’s spiritual realization. Hence, the practitioners who are able to produce sacred relics after death are understood to have attained spiritual mastery that occurred at the time of death. The *ringsel* are perceived as one way in which Buddhist practitioners transmogrify their connection with their students into material form (see Karthar, 2010; Tidwell, 2024; Zivkovic, 2014, for different kinds of *ringsel* in Tibetan Buddhist culture).

## Interaction with Biomedical Doctors

Local residents as well as the media flocked to the monastery, and within no time, the news of a “miraculous monk in meditation” spread out. Lobsang Rinpoche’s feat of continued meditation even after his (clinical) death had reinforced his colleagues’ and students’ faith in his spiritual insight. It amazed local communities—both Tibetan and Indian—to witness something that they had only heard about from spiritual teachers or read in ancient texts. Likewise, the news baffled local doctors and scientists. In the following days, delegates from hospitals and scientific organizations came to see Lobsang Rinpoche’s dead yet “meditative” body.

On the fourteenth day of Lobsang Rinpoche’s *tukdam*, a team of doctors from Karnataka Lingayat Education Society (KLES) Prabhakar Kore Hospital came to the monastery to see Rinpoche’s body. KLES is one of the largest hospitals in north Karnataka state and is approximately 90 miles north-west of Mundgod. The team was led by a doctor who attended Lobsang Rinpoche at the time of his death and apparently signed his death certificate. The doctor told us that they had been curious when they read about the meditative state of Lobsang Rinpoche in the local news article and wanted to see it for themselves. I escorted them into the room where Lobsang Rinpoche was lying. As the doctor got near the bed, he stood there staring for a few minutes and then got all teared up. Once outside, the doctor said it was hard for him to believe what he saw: “The person who died in front of me two weeks ago looks every bit alive and has no signs of decomposition. This is unbelievable.” Moreover, the doctor said he was overwhelmed by emotion because he had heard of such spiritual practice but seeing it in person was something he had never imagined.

**Fig. 2:**
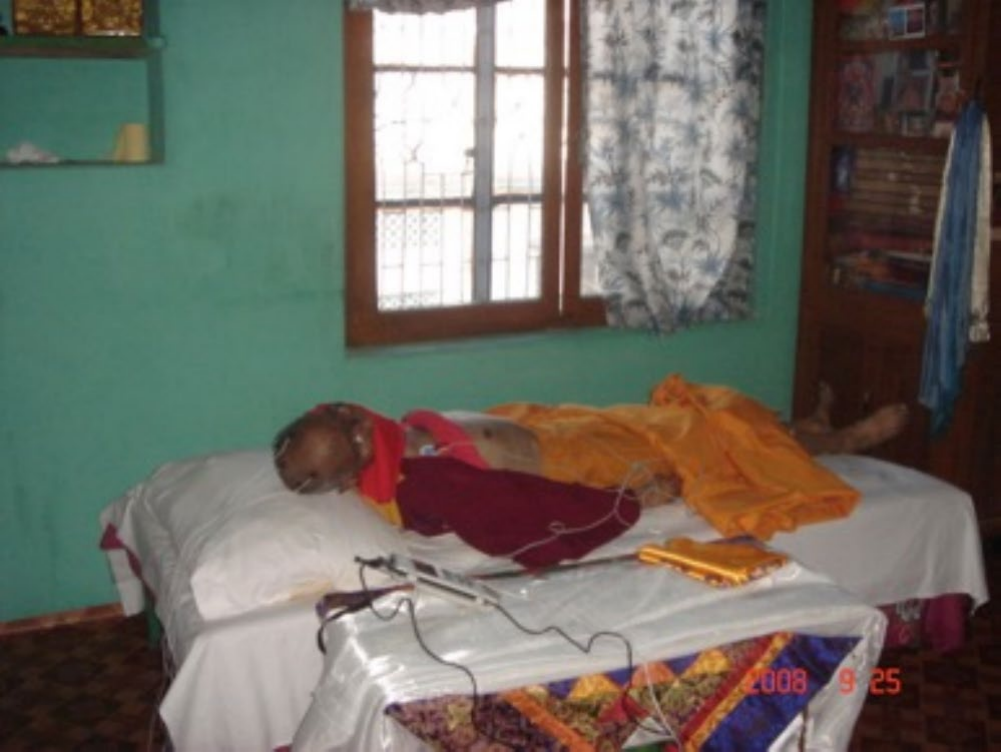
Lobsang Rinpoche in his thirteenth day of *tukdam* after clinical death

As we were talking, I realized I had a similar feeling—a deep sense of respect for Lobsang Rinpoche and curiosity about a cultural phenomenon that seemed extraordinary. Hence, in the midst of everyone experiencing a collective state of reverence and amazement at the meditator’s feat, it made me wonder what the meditator—Lobsang Rinpoche, in this case—must be experiencing while in *tukdam*. Why would he choose to dedicate his life to prepare for a particular moment at the time of dying to engage in such a complex contemplative technique of this nature? What is the purpose of staying in *tukdam*? And how is one able to stay in *tukdam*? (see Fig. 2).

## *Tukdam*: A Nexus of Supreme Technique and Sublime Knowledge

I thought that the best way to respond to my curiosity would be to talk to monks at the monastery, who, besides possessing a certain level of academic authority, are adept practitioners. So, my colleague and I contacted Loden and Choesang, and they helped to arrange a meeting with Geshe^4^ Namgyal Wangchen. Geshe Wangchen was popular among the monastic community owing to his scholastic credentials as well as being an extremely open and kind teacher. He had spent a decade in London as a teacher and thus was fluent in English. Though excited, I was a little intimidated when we went to see him.

Geshe Wangchen greeted us with a warm smile, and said, “They told me you two are trained in both the Western and Tibetan medical systems, so this is great because I am taking both Tibetan and Western medicine.” Emphasizing the benefit of integrating Western and Tibetan medicine, he burst into a mild laughter and said he also had questions for us related to his health and modern science. Geshe Wangchen had been diabetic for many years and was recently diagnosed with hypertension. We discussed the management of diabetes from both Tibetan and Western medical perspectives through diet, behavior, and medication. He also seemed very interested in the intersection of Buddhism and science and talked about a book he was working on focused on the philosophy and psychology of Buddhism and modern science.

As we started to talk about the scientific study of *tukdam*, Geshe Wangchen said, “I know Lobsang Rinpoche well and worked closely with him. He was a wonderful scholar and an adept practitioner.” With a little hesitation, I asked him the purpose of *tukdam* for a practitioner like Lobsang Rinpoche, who was highly respected and had little to prove in terms of his spiritual practice. I realized later as I spent more time in the field though that *tukdam* has nothing to do with ‘proving’ anything. It was good to know because I remember wondering if students and other followers’ expectations that their teachers should stay in *tukdam* would put pressure on those practitioners to do so.

Zivkovic’s (2010) important work on spiritual biographies of Buddhist practitioners showed the significance of having control over body, mind, and consciousness at the time of death. She noted that understanding death and controlling the process of dying hold the key to assessing the spiritual achievement of Buddhist lamas as illustrated through one of her interlocutor’s remarks: “It is after death that we can truly know a lama and their ability” (175). Geshe Wangchen, however, was clear that *tukdam* should never be employed as a measure of a practitioner’s spiritual achievement. He also wanted us to know that not everyone whose body does not decompose right away is in the state of *tukdam*. He noted:

*Tukdam* is a powerful contemplative technique, but it is also important to differentiate *tukdam* propelled by strong emotions from one that is spiritually motivated based on years of study and practice. *Tukdam* motivated by strong anger or attachment generally lasts two to three days whereas the one facilitated by spiritual tantric endeavor could continue for weeks or months. Anyone might stay in a *tukdam-*like state; however, there is a difference between a person dying with lots of attachment or anger and a practitioner who dies confidently with complete clarity about the process of dying and how to use a dying stage to coincide with the subtlest states of mind.^5^

Geshe Wangchen’s emphasis on differentiating *tukdam* from a *tukdam*-like state or pseudo-*tukdam* was crucially informative because I would later come across several occasions during my fieldwork where the line between someone being in *tukdam* and not was blurry, and in some cases, complicated by various related sociocultural factors. There was a case where a deceased senior monk’s body was showing all the signs of decomposition, but his students were adamant about his continued meditation. At that time, Geshe Wangchen’s assertion about the length of *tukdam* was helpful for me as well as others at the monastery when I explained to them both an adept practitioner’s perspective like that of Geshe Wangchen (see also, Dalai Lama XVI, 2003, p. 150; Lati & Hopkins, 1979, p. 45) and the biomedical interpretation of post-death physiological changes (see Tidwell et al., 2024, for further details).

However, my insatiable curiosity about how one can stay in *tukdam* led me to meet with a well-known senior teacher, Geshe Lobsang Dukdha, during my fieldwork in the spring of 2017. Geshe Dukdha is one of the senior teachers at the Gyudmey Tantric School in a small Tibetan refugee settlement called Hunsur. Hunsur is located south of Mundgod, 260 miles from where my main research field site was based.

Geshe Dukdha was in his early fifties and unusually tall for a Tibetan. He had a serious facial expression that could be intimidating for people who do not know him well. I remember meeting him briefly during my pre-dissertation research in 2014. At that time, he was supportive of my research, encouraging me by saying that my work would not only help further academic understandings, but also provide personal (spiritual) growth vis-à-vis a deeper understanding of life and death. I was delighted to see him again given this earlier encounter. I had prepared a set of questions for him but started by asking how one stays in *tukdam* and the reason it is important for practitioners who choose to stay in *tukdam*. Geshe Dukdha posited three ways in which one can stay in *tukdam*: (1) based on one’s life-long practice; (2) based on an initiation one received from high lamas and being profoundly committed to the vows taken during those teachings; and (3) through a deep sense of reverence and faith toward one’s spiritual teacher (for example, the Dalai Lama) at the time of dying.

“*Tukdam* is really about gradually suspending one’s sensory consciousness and then, being focused, contemplating at the subtle, and very subtle levels of consciousness,” Geshe Dukdha said. “It is similar to falling asleep when one’s sensory faculties slowly fade away and after a while, what one has is just subtle consciousness.” “Likewise,” he continued, “When one engages in meditation to slow down gross consciousness, the primary approach is to simulate a sleep-like state, or, if possible, the stages of dying, and get to the subtlest form of consciousness to examine oneself and one’s relationship to others by contemplating *tong nyi”* (*stong nyid*, lit. emptiness). Dukdha further added:

*Tukdam,* therefore, becomes an extremely important time and context to investigate ultimate truth^6^ in a most effective manner. It is a meditative technique that helps a practitioner to contemplate and become familiarized with it, and therefore *tong nyi*. One engages in *tukdam* at the stage of *chiwé ösel* (*’chi ba’i ’od gsal*; the clear light of death) at the subtlest level of consciousness during the process of dying. In the Clear Light of Death, one’s level of mental faculty is hyper amplified, and attention, completely undivided.

As per Geluk tradition in Tibetan Buddhism, the technique of meditating at the subtlest level of consciousness requires years of dedicated study and self-investigative modes of practice (see Tidwell, 2024, for different Tibetan Buddhist traditions approaches to tukdam). Thus, it is regarded as a supreme method among all techniques by Tibetan Buddhist practitioners. This is so because when the sensory organs associated with the brain cease to function—either while simulating a dying process or at the time of dying—the subtlest mind is free from any distraction that is usually caused by sensory reaction to stimuli. This is particularly true at the stage of dying called the Clear Light of Death when the subtlest level of consciousness is so clear and sharp that it can realize the nature of whatever it engages. In Tibetan Buddhist philosophy, consciousness in its natural state is defined as luminous and knowing (Dalai Lama XIV, 2017). Luminous means the ability of consciousness to reveal and disclose; and knowing relates to the ability of consciousness to perceive and apprehend what appears (Thompson, 2015).

Tibetan Buddhist practitioners assert that employing meditative techniques to contemplate emptiness, or *tong nyi*, at this specific point in the dying process can produce supreme insight. Geshe Samten Gyatso, one of the senior practitioners of Gomang Monastic School, affirmed the importance of studying the eight stages of dying. “Knowledge about the stages of dying,” Geshe Gyatso emphasized, “is imperative for anyone, whether they are Buddhist practitioners, healthcare personnel, or lay people.” The eight stages of dying go from a coarse or gross physical and mental state, to the subtle and then to the very subtle. Below, I provide a summary of his description.

## Eight Stages of Dying

Tibetans who have studied and are familiar with the process of dying employ this understanding at the time of their own death, as well as while caring for dying people to help prepare them for impending death. As the body disintegrates, stages of dying are marked by external physiological changes, weakening of sensory faculties, and internal experiences.

In Tibetan Buddhist practice, the *first stage* of manifestation is the earth element, one of the five sources^7^ that comprise the human body, slowly diminishing. This leads to the external signs of the body becoming thin yet heavy, the loosening of joints, the loss of eyesight, and difficulty in opening or closing the eyes. The internal experience is that of sinking into the earth and seeing a mirage. In the *second stage*, as the water element diminishes, the external signs include the decedent losing control of bodily fluids; saliva, sweat, and urine drying up extensively; and the auditory faculty becoming weak. Internally, the dying person would perceive the appearance of smoke everywhere. In the *third stage*, as the fire element diminishes, the warmth of the body fades; dietary intake can no longer be digested; memory declines, and the sense of smell dissipates. The dying person would have an experience likened to seeing fireflies in the sky. In the *fourth stage*, as the air element diminishes, the external signs include having trouble breathing, hallucination, losing the sense of touch and taste, experiencing the thickening, and shortening of one’s tongue, and gradually experiencing the cessation of breath. The person would perceive the appearance of a spluttering butter lamp. At this stage, the dying person is clinically dead, and the gross consciousness dissolves, making the subtle consciousness more prominent.

In the *fifth stage*, the White Appearance (*snang ba dkar lam pa*) manifests at the prominence at the middle of the crown of the head connected to the central channel of the body loosens and the white drop or essence from our father descends toward the heart. This leads to the phenomenological experience of proceeding along a white path like the clear autumn sky permeated by moonlight. In the *sixth stage*, the Red Increase (*mched pa dmar lam pa*) appears, where the navel point related to the sexual organ loosens and the red drop or essence from our mother ascends toward the heart. This leads to the internal experience of perceiving an autumn sky pervaded by the sunset. The *seventh stage* is known as the Black Near Attainment (*nyer thob nag lam pa*), where the white and red drops unite at the heart and cause the dying person to experience vivid blackness. Finally, the *eighth stage* is known as the Clear Light of Death (’c*hi ba’i ’od gsal*), where the merged white and red integrated essence dissolves into an indestructible drop at the heart and the subtle wind, or *loong*^8^ (*rlung*), energy dissolves into the subtlest mind. At this stage, the mind is clear, and receptive to the highest level of mental function (see Lodoe, 1995, pp. 21–23 and Lati and Hopkins 1979 for a detailed explanation of the eight stages of dying).

After Geshe Gyatso explained these eight stages, I asked with intrigue, “Do all dying people go through these eight stages, or just Buddhist practitioners?” Geshe Gyatso gave me an affirming smile. “I am glad you asked this,” he said. “This is important to know because being calm, mindful, and attentive to what is happening while dying is crucial for a dying person. It is crucial because although everyone goes through these dying stages, only those dying with a calm mind are able to be aware of these subtle stages, particularly, the very subtle stage—the Clear Light of Death state—when a person can employ that opportunity to meditate.” I observed regularly during my fieldwork that people dying calmly were less anxious generally and more positive as things unfolded in the last phases of their lives.^9^

## Investigating the Ultimate Truth

During my fieldwork, one theme that arose frequently during my interviews and interactions with senior Buddhist teachers and monks at monasteries was the importance of being in touch with the true nature of “reality.” I was not fully aware of the context in which my interlocutors understood reality in the beginning but as I spent more time with the monks, particularly with my field supervisor, Geshe Phuntsok Dhondup, I realized that their “reality” referred to the ultimate truth. They would contend that the ultimate truth is different from the reality we usually refer to, which in Buddhist philosophy is interpreted as conventional truth.

The two truths—conventional truth (*kun rdzob bden pa*) and ultimate truth (*don dam bden pa*)—comprise one of the main ontological doctrines in Buddhist philosophy and underpin the socio-moral fabric of Buddhist cultural communities (see Cameron & Namdul, 2020 for further explanation). For instance, the reality that the source of happiness depends upon external factors, or the reality of an intrinsic self is perceived as reality that is not fully examined (see Ozawa-de Silva, 2006; Tsondu & Dodson-Lavelle, 2009 for further elaboration on reality from the Buddhist perspective). The intriguing aspect of viewing reality based on two truths for lay Tibetans is that none of our mundane reality is more or less real, rather it is about having an agency to analyze and infer the mechanism and outcome of one’s behavior and thus make informed life choices. I remember one of my first interviews during my fieldwork, Geshe Dhondup started by saying, “All the problems we are facing are due to our actions that are not aligned with reality.” He further added that the reality that is intellectually investigated and understood has a profoundly positive impact on one’s well-being:

All the sufferings in our life can be traced to our failure to differentiate between conventional and ultimate reality. Conventional reality is based strongly on our daily life triggered by sensory emotions, such as seeing something beautiful, listening to nice music, or being physically attracted to someone; and these phenomena might be conceived as a source of happiness but could lead to suffering. Conversely, being able to control these emotions by examining and understanding both the immediate and long-term outcome of our behaviors allows one to be happy without relying on external conditions.

Geshe Dhondup had a distinct way of conveying complex Buddhist concepts as easily relatable and motivating. He had a humble, warm smile when he talked, and his facial expression carried an unequivocal sense of genuine care and concern. I realized over time that I was not the only person who felt his positive ambience.

Geshe Dhondup emphasized the way Buddhist practitioners would use their amplified consciousness to gain deeper knowledge about their minds, and importantly, the way adept practitioners would employ their focused mind to meditate on emptiness. “Contemplating emptiness and being able to comprehend the operation of emptiness,” he says, “is the essence of knowledge.” And as usual, with a child-like smile, he further highlighted the importance of realizing emptiness by saying, “Just like you have *rinchen rilbu*^10^ [precious pills] in Tibetan medical practice that are considered more efficacious than other pills in treating illness, the knowledge and insights one gains from contemplating emptiness are like the king of medicine for curing our mental suffering.” When I asked him about the relationship between gaining deeper insight into emptiness and one’s preparation for death, Geshe Dhondup said:

Emptiness is one of the key concepts of Buddhist philosophy. Although emptiness is explained at different levels, it essentially asserts that there is no independent or intrinsic self. Everything is dependent upon multiple factors. For instance, if it is not for the love, care, nurturing, and support of our parents and everyone in our life, we would not have survived. This is the reality we all need to be in touch with. Likewise, death is another reality of our life. One of the reasons we are so scared and anxious about death is that we are not able to accept the reality that we all will die and that it could happen at any moment of our life.

## Conclusion of Lobsang Rinpoche’s *Tukdam*

It was the seventeenth day since Lobsang Rinpoche had entered *tukdam* when, one evening as we were just getting done setting up our devices and attaching electrodes to Lobsang Rinpoche’s head and chest, Jampa Rinpoche summoned us. He said he had dreamt of certain signs, which might indicate the conclusion of *tukdam*. He wanted us to be prepared in case Lobsang Rinpoche would conclude his *tukdam*. Upon us asking, Jampa Rinpoche expounded upon the signs associated with the conclusion of *tukdam*:

Usually, the meditator will let go of control over the subtle *loong* and consciousness. When that happens, we start seeing physiological changes, such as discoloration, putrefaction, loss of body heat, etcetera. I dreamt Rinpoche’s body had some smell and there were changes happening in his body. Also, when *tukdam* is about to be concluded, the weather gets cooler, clouds start forming, and a rainbow can appear.

Jampa Rinpoche was right. The next day, we had unusual weather. It got unbearably hot in the morning, followed by a strong wind, red dry dust churned up in the air, and a heavy downpour for almost an hour. By noon, the rain had stopped, the temperature cooled, and we had a clear sky where a rainbow appeared. Loden, one of the main attendants of Lobsang Rinpoche, came to us, and with seemingly mixed feelings, he told us, “It seems like Rinpoche has released his subtle consciousness,” meaning, Lobsang Rinpoche had concluded his *tukdam* state.

Loden mentioned there was a little discharge of semi-red fluid from Rinpoche’s nostril. It is generally observed that a light-red fluid and a light-white fluid will be released from the nostrils and genitals, respectively, when a practitioner releases their subtlest consciousness at the conclusion of *tukdam* (see Tidwell, 2024). By the time we went to the room, Rinpoche’s body had started to change, already showing signs of decomposition. It was quite astonishing to see how fast Rinpoche’s body started to change as he concluded his meditation. The radiance on his face was quickly vanishing, his skin color changed, especially under the chin, behind the ears, under the arms, and some parts of legs; and fluid started to be retained in his back region and legs.

Everyone was preparing for the cremation but suddenly, everything changed. Loden and Choesang were running around arranging a new casket made especially of oak. By late afternoon, Lobsang Rinpoche’s body had been washed, ceremonies performed, and a truck filled with raw sea salt showed up by the gate of Rinpoche’s house. As I was wondering what was happening, Loden came over and told me that the Dalai Lama had specifically directed them to mummify Lobsang Rinpoche’s body. I was told that mummification of a body is usually done for select practitioners to continue their connection with their students as well as to inspire followers to be committed to their practices (see Zivkovic, 2014 for further description).

When my colleague and I left Drepung Loseling, the monks were in the process of mummifying Lobsang Rinpoche’s body employing the traditional method of using sea salt. Along with Rinpoche’s body, the attendants filled the casket with sea salt, changing the salt every three to four days to a week. It was an extraordinary experience for me to see someone in *tukdam* so closely and then to be part of the whole milieu that was generated during and after *tukdam*. This cultural phenomenon raises an interesting question about if someone in *tukdam* is dead or dying. For instance, Lobsang Rinpoche was declared dead at the hospital based on the cessation of his brain and cardiopulmonary functions. However, for Rinpoche’s followers and others around him, he was in the process of dying even after these physiological functions had ceased, for his (subtle) consciousness had not yet left his body. Such phenomena challenge biomedicine’s determination of death, which strictly relies on biological markers to be observed and measured (see Namdul, 2019, 2021 for further description).

I distinctly remember going back to Dharamsala with a conviction that I wanted to know more about *tukdam,* a cultural phenomenon that uses death as a vantage point in one’s investigation of self and one’s relationship to others. I was particularly intrigued by the unique cultural phenomenon where death is not only viewed as a heuristic ground in investigating and shaping the socio-moral fabric of life, but also in cultivating profound compassion and resilience in the face of an existential fear of death. It made me question the very purpose of death and the role conceptions about death play in how people live.

## The Purpose of Death

Both my early encounter of Lobsang Rinpoche in *tukdam* and ethnographic fieldwork of studying how Tibetans care for dying people present dilemmas that not only challenge the way death is conceived, but also the role death plays in shaping and reinforcing cultural values and beliefs (see Lott, 2024; Author, 2021, for similar arguments). The question of “what is the purpose of death” is inevitable considering the way death and dying are understood and applied in everyday life as well as in spiritual practice among Tibetans. Many of my interlocuters, while explaining what death is, also talked about the “utility” of death for spiritual practice (see Dalai Lama XIV, 2003; Karthar, 2010; Author 2021).

Likewise, when I asked my interlocuters, “How do you conceive death?” they would respond with varied responses based on their education background or age, but one overarching response was, “Death is a part of life. Whoever is born has to die.” Some of them would elaborate further about distinguishing between life and death by saying, “Death is a moment when consciousness leaves the body.” My field supervisor Geshe Dhondup summed up the cultural significance of death by relating death to a constant reminder of the reality with which we should always be in sync. He said:

The reality of death is important for us to remind ourselves not only of the constant changes occurring in our body and outside in the environment, but also to tame our insatiable desire and attachment. Similarly, for advanced practitioners, stages of dying are crucial moments for further advancement of their spiritual practice. In that sense, death and dying holds an important place in worldly life as well as spiritual practice.

## Conclusion

The treatment of death and dying reflects fundamental components of a cultural practice and its impact on the way people view death and care for dying people. Tibetan Buddhist culture has a specific way of dealing with death, but its uniqueness is the epistemological “status” of death. Death and dying are feared, not because they are unknown since death ruptures the familiar continuity of life, but because they are intimately understood. As such, they can be a source of flourishing, and if used particularly well, the grounds for enlightenment. I have observed that practitioners—both monastics and laypeople—employ death to extrapolate meaning in combating limitless desire and attachment fueled by an ignorant mind. Likewise, dying is used in generating an altered consciousness that presents a hyper-receptive agency in investigating, realizing, and embodying emptiness. Such mental engagement at the subtlest level with a clear motivation and purpose, I argue, amounts to a mechanism to alchemize the existential fear and suffering of death to produce existential clarity and joy (see Namdul, 2021 for further explanation).

The ethnographic account of Lobsang Nyima Rinpoche’s *tukdam* illustrates a Tibetan Buddhist practitioner’s engagement with the subtlest mind at the time of dying. Even though *tukdam* is practiced by advanced Buddhist practitioners, the philosophy and psychology that underpin *tukdam* inform the core components of cultural models that deal with death and care for the dying among Tibetans. Furthermore, this article shows the significance of death as a Tibetan Buddhist cultural-reference that offers a moral heuristic ground for adaptive methods in transforming orientations to self and other and in cultivating “heart power” or mental strength in the face of traumatic experiences and existential threat, such as death. This article thus articulates why Tibetan Buddhist practitioners emphasize a strong association between a true understanding of self and sustained happiness. Likewise, the article responds to a key questions of: why do Tibetans believe that meditating on death is crucial to a sense of well-being in their daily experience? And what is the relation between the temporalities of the meditated death and that of the day-to-day life? Using *tukdam* as a framework that provides a fundamental structure to Tibetan cultural notions and practices of death and dying, this article proposes that *tukdam* symbolizes the way death and dying is assumed to be approached beyond adept practitioners, and thereby, provide a model for an “ideal” death that guides approaches for oneself and others.

## Data Availability

Research Data Policy: there are no data repository to be shared. The data cannot be shared because, (a) this is an ethnographic study and the interviews of the interlocutors cannot be de-identified; (b) given the study participants being Tibetan refugees in exile, there are potential risks for their family members in Tibet.

## Footnotes

1 Rinpoche (*rin po che*) in Tibetan literally means “precious one” or “jewel;” and in the context of Tibetan Buddhism, it is employed to refer to a reincarnate lama or highly respected religious teacher. Note: all informant names are used by consent unless otherwise specified.

2 Gaden (also transliterated as Ganden) Tripa (*dga’ ldan khri pa*) is the title of the spiritual leader of the Geluk school of Tibetan Buddhism. Unlike the system of recognizing reincarnations, the Gaden Tripa is an appointed position, based solely on the intellectual and spiritual merit of a practitioner as assessed by the Governing Body including the Dalai Lama.

3 For other ethnographic accounts, see Coleman (2017, 2025); Lott 2024; Tidwell 2024.

4 Geshe (*dge bshes*) or Geshema (*dge bshes ma*) is a Tibetan Buddhist academic degree for monks and nuns, respectively. Geshe Lharam (*dge bshes lha ram*) is the highest degree in Geluk tradition.

5 All conversations occurred in Tibetan and were translated by the author unless otherwise noted.

6 Ultimate truth, which is one of the Two Truths in Buddhist philosophy, will be explained further below.

7 Five sources (*’byung ba lnga*, pronounced *jungwa nga*, also translated as “five elements”) is one of the fundamental theories related to the formation of the physical body in Tibetan medicine and Tibetan Buddhist tantric practice. The five sources are: earth, water, fire, wind, and space.

8 Loong (*rlung*)*,* is one of the three principal energies in Tibetan medicine, responsible for every moment in the body, akin to the neuroendocrine system in Western biomedicine. Since *loong* is understood to have a bi-directional relationship with the mind, it is explained in length in Tibetan Buddhist tantric practice. Further, *loong*, in this context, can also be articulated as breath-related energy.

9 Robert Desjarlais related a similar understanding of the importance of one’s state of mind at the time of death in his interactions with those of the Yolmo Buddhist community (2016).

10 *Rinchen rilbu* (*rin chen ril bu*), comprised multiple herbs, precious gems and metals, is considered the most potent class of Tibetan medicinal formulations, and is used for treating various ailments. It is also prescribed as a prophylactic and rejuvenating medicine (see Czaja, 2016 for a detailed description).
